# A prospective study of nomogram-based adaptation of prostate radiotherapy target volumes

**DOI:** 10.1186/s13014-015-0545-y

**Published:** 2015-11-25

**Authors:** Raymond Wu, Hannah Woodford, Anne Capp, Perry Hunter, Gary Cowin, Keen-Hun Tai, Paul L. Nguyen, Peter Chong, Jarad Martin

**Affiliations:** Department of Radiation Oncology, Calvary Mater Newcastle, Edith Street, Waratah, NSW 2298 Australia; University of Newcastle, School of Medicine and Public Health, University Drive, Callaghan, NSW 2308 Australia; University of Queensland, The Centre for Advanced Imaging, Building 57, Research Road, St Lucia, QLD 4072 Australia; Department of Radiation Oncology, Peter MacCallum Cancer Institute, St Andrews Place, East Melbourne, VIC 3002 Australia; Brigham and Women’s Hospital, Radiation Oncology, 75 Francis Street, Boston, MA 02115 USA; Sky Central East, Level 3, Suite 2, 20 Smart Street, Charlestown, NSW 2290 Australia

**Keywords:** Prostatic neoplasms, Radiotherapy, Nomograms

## Abstract

**Background:**

A prospective clinical trial was conducted to evaluate the feasibility of a novel approach to the treatment of patients with high risk prostate cancer (HRPC) through the use of a nomogram to tailor radiotherapy target volumes.

**Methods:**

Twenty seven subjects with HRPC were treated with a mildly hypofractionated radiotherapy regimen using image-guided IMRT technique between Jun/2013-Jan/2015.

A set of validated prognostic factors were inputted into the Memorial-Sloan-Kettering Cancer Center (MSKCC) prostate cancer nomogram to estimate risk of loco-regional spread (LRS). The nomogram risk estimates for extra-capsular extension (ECE), seminal vesicles involvement (SVI), and pelvic lymph nodes involvement (LNI) were used to adapt radiotherapy treatment volumes based on a risk threshold of ≥15 % in all cases. A planning guide was used to delineate target volumes and organs at risk (OAR). Up to three dose levels were administered over 28 fractions; 70Gy for gross disease in the prostate +/− seminal vesicles (2.5Gy/fraction), 61.6Gy for subclinical peri-prostatic disease (2.2Gy/fraction) and 50.4Gy to pelvic nodes (1.8Gy/fraction).

Data regarding protocol adherence, nomogram use, radiotherapy dose distribution, and acute toxicity were collected.

**Results:**

*Nomogram use*

100 % of patients were treated for ECE, 88.9 % for SVI, and 70.4 % for LNI. The three areas at risk of LRS were appropriately treated according to the study protocol in 98.8 % cases. The MSKCC nomogram estimates for LRS differed significantly between the time of recruitment and analysis.

*Contouring protocol compliance*

Compliance with the trial contouring protocol for up to seven target volumes was 93.0 % (159/171). Compliance with protocol for small bowel contouring was poor (59.3 %).

*Dose constraints compliance*

Compliance with dose constraints for target volumes was 97.4 % (191/196). Compliance with dose constraints for OAR was 88.2 % (285/323).

*Acute toxicity*

There were no grade 3 acute toxicities observed. 20/27 (74.1 %) and 6/27 (22.2 %) patients experienced a grade 2 genitourinary and gastrointestinal toxicity respectively.

**Conclusions:**

We have demonstrated the feasibility of this novel risk-adapted radiation treatment protocol for HRPC. This study has identified key learning points regarding this approach, including the importance of standardization and updating of risk quantification tools, and the utility of an observer to verify their correct use.

**Trial registration:**

ClincialTrials.gov identifier NCT01418040.

Hunter New England Human Research Ethics Committee (HNEHREC) reference number 12/08/15/4.02

## Background

Radiotherapy (RT) has been shown to independently improve overall survival for men with high risk prostate cancer (HRPC) managed with androgen deprivation therapy (ADT) [1, 2]. The traditional approach to radiotherapy for HRPC is to treat the prostate alone. However, there is extensive surgical pathological literature demonstrating the risk of subclinical disease infiltration of HRPC into pelvic lymph nodes (PLN), seminal vesicles (SV), and in an extra-prostatic distribution [3–5]. With the increased uptake of intensity modulated radiotherapy (IMRT), there is a potential opportunity to tailor treatment to such areas at significant risk of loco-regional spread (LRS) rather than managing all men with HRPC in an identical manner. In other sub-sites, for example in the treatment of mucosal squamous cell carcinoma of the head and neck (HNSCC), this treatment approach has long been accepted as standard of care.

Uncertainty regarding the role of whole pelvis radiotherapy (WPRT) in high risk prostate cancer is reflected in various clinical guidelines, in which the elective treatment of pelvic nodes is left up to the treating clinician’s discretion [6, 7]. Two randomized controlled trials (RTOG 9413 and GETUG-01) have failed to convincingly demonstrate improvement in progression free survival with the use of WPRT versus prostate-only treatment [8, 9], although later results from the RTOG study show improved biochemical control in a subset of patients receiving neo-adjuvant hormonal therapy. Reasons for a lack of benefit from WPRT have been described, including insufficient radiation dose, inadequate coverage of at-risk nodes, and poorly targeted patient selection [10, 11]. Despite the lack of level I evidence for WPRT, this practice has been incorporated into the standard treatment of HRPC in multiple practice-defining randomized controlled trials (RCT) [12–14].

WPRT in this setting is not without its risks; there is mixed evidence to suggest increased acute and late grade 3 gastrointestinal toxicity and decreased bowel quality of life [8, 9, 15, 16]. Despite reductions in dose to critical structures and late GI adverse effects achieved through the use of IMRT over 3D-conformal RT [17, 18], WPRT is still likely to result in increased toxicity compared to treatment of the prostate alone. It is therefore important to reserve the use of WPRT, and to a lesser extent irradiation of the SV and peri-prostatic regions, for those patients that are most likely to experience improved tumour control outcomes.

We conducted a prospective clinical trial to assess the feasibility and tolerability of a hypofractionated radiotherapy treatment protocol for HRPC that employed the use of a widely accessible and externally validated online nomogram to estimate risk of LRS and accordingly adapt delineation of target volumes.

## Methods

### Study design and participants

This prospective phase two single institution study enrolled patients with high-risk prostate cancer for 18 months (Jan 2013-June 2014). Patients were eligible for the study if they met the following inclusion criteria: histologically confirmed adenocarcinoma of the prostate, high risk disease (defined by any one of baseline prostate-specific antigen (PSA) ≥ 20 μg/L, Gleason Score (GS) 8–10 and/or clinical stage T3-T4), and conventional staging imaging negative for distant metastases (technetium-99 m whole body bone scan and CT of abdomen and pelvis). Exclusion criteria included: previous pelvic radiotherapy, history of prior malignancy within the last 5 years (excluding non-melanomatous skin cancers), Eastern Cooperative Oncology Group (ECOG) performance status ≥ 2, or any contraindication to insertion of intra-prostatic fiducial markers or planning MRI prior to RT simulation. All patients were administered a total of 18 months of ADT in the form of Leuprorelin 22.5 mg every 3 months.

All patients gave written informed consent. The study was reviewed and approved by the Hunter and New England Human Research Ethics Committee (HNEHREC Ref: 12/08/15/4.02). The trial was registered with ClincialTrials.gov (identifier NCT01418040).

### Nomogram use

A set of parameters (age, PSA, tumour GS, clinical stage and percentage of positive biopsies) were inputted into the Memorial-Sloan-Kettering Cancer Center (MSKCC) prostate cancer nomogram [19] prior to radiotherapy and again at time of analysis to estimate risk of LRS. The pre-RT nomogram risk estimates for extra-capsular extension (ECE), seminal vesicles involvement (SVI), and pelvic lymph nodes involvement (LNI) were used to adapt radiotherapy treatment volumes based on a risk threshold of ≥15 % in all cases.

### Simulation and planning protocol

Radiotherapy commenced after 6 months of neo-adjuvant ADT, in keeping with the results from the TROG 96.01 randomised trial showing superiority of this duration verses 3 months or no ADT [20]. Following insertion of three intra-prostatic gold fiducials, all patients underwent CT simulation (Aquilion LB TSX-201A, Toshiba Medical Systems Corporation) in the supine position with customized immobilization. Patients were instructed to have a comfortably full bladder and an empty rectum for simulation and treatment. A 3-tesla non-contrast planning MRI scan (MAGNETOM Skyra, Siemens) using 2 mm slices and T2 weighting was completed on the same day and co-registered with the simulation CT by matching to the fiducial markers.

A standardized planning guide was developed and used to direct contouring of target volumes and organs at risk (OAR) on the CT and MRI imaging. Target volumes were contoured as listed in Table 1. Elective irradiation of extra-capsular disease extension, proximal seminal vesicles, and/or pelvic lymph nodes was completed if the risk of involvement of each respective region exceeded 15 %, as estimated by the MSKCC nomogram.

**Table 1:** Contouring protocol for target volumes

Structure	Contouring protocol	Condition
CTV_P_	Prostate as defined using CT and MRI imaging + any extra-prostatic extension as noted on examination or pre-ADT imaging	All patients
CTV_ECE_	3 mm isotropic margin from CTV_P_, excluding overlap with rectum	If ECE risk ≥ 15 %
CTV_SVI_	Entire bilateral seminal vesicles (only contoured if known seminal vesicle involvement)	If SV involved
CTV_SVA_	Proximal 20 mm of SV, measured obliquely along long axis of SV (only contoured for adjuvant treatment of seminal vesicles)	If SVI risk ≥ 15 %
CTV_LN_	Pelvic nodes: 7 mm margin around obturator, pre-sacral, and external and internal iliac vessels contoured as per RTOG consensus guidelines [21], up to 10 mm inferior to the sacral promontory)	If LNI risk ≥ 15 %
PTV70	If no SV involvement: 5 mm margin around CTV_P_ If SV involvement: 5 mm margin around CTV_P_ + 7 mm margin around CTV_SVI_ anteriorly and posteriorly and 5 mm otherwise	All patients
PTV61.6	If SV involvement or SVI risk <15 %: 5 mm margin around CTV_ECE_ If no SV involvement and SVI risk ≥15 %: 5 mm margin around CTV_ECE_ + 7 mm margin around CTV_SVA_ anteriorly and posteriorly and 5 mm otherwise	If CTV_ECE_ or CTV_SVA_ contoured
PTV50.4	5 mm margin around CTV_LN_	If CTV_LN_ contoured

The rectum, bladder, neck of femur, small bowel and penile bulb were contoured as organs at risk (OAR). The rectum was contoured as a solid organ from the ano-rectal junction to the recto-sigmoid flexure. The entire bladder was also contoured as a solid organ. The small bowel was contoured as any visible small bowel as well as peritoneal contents within 8 mm of the superior margin of the PTV. This volume was expanded 3 mm in all directions to create a small bowel planning organ at risk volume (PRV).

### Radiotherapy technique

Radiotherapy was administered over 28 daily fractions, given five fractions per week using an image-guided dynamic IMRT technique. Pre-treatment image guidance was conducted using matching of kilovoltage imaging to the three intra-prostatic gold fiducial markers with a 1 mm action threshold for a translational shift. Extrapolating from the HNSCC literature, and given the expectation of reduced clonogen density in imaging-negative areas, reduced radiotherapy dosing was administered to elective regions. Up to three dose levels were treated in 28 fractions using a simultaneous integrated boost:

- All patients received radiotherapy to the prostate +/− seminal vesicles (if grossly involved on clinical examination or MRI) to a dose of 70 Gy (2.5 Gy per fraction).
- If the nomogram estimate for ECE ≥15 %, an additional volume (formed by a 3 mm isotropic expansion from the prostate excluding overlap with the rectum) was treated to 61.6 Gy (2.2 Gy per fraction).
- If the nomogram estimate for SVI ≥15 %, the proximal 20 mm of the seminal vesicles was treated to 61.6 Gy (2.2 Gy per fraction).
- If the nomogram estimate for LNI ≥15 %, the pelvic lymph nodes were treated to 50.4 Gy (1.8 Gy per fraction), contoured according to RTOG consensus guidelines [21] with a modified upper border of 10 mm inferior to the sacral promontory.

### Dose constraints

Planning objectives and field arrangement were optimized to achieve the best dosimetry to satisfy dose constraints for target volumes and OAR (listed in Table 2). All planning was performed using the Eclipse Treatment Planning System v12 (Varian Medical Systems). In particular, dosing to the PTVs aimed to deliver 100 % of the prescribed dose to 98 % of the target volume as per ICRU 83 [22].

**Table 2:** Dose constraints for target volumes and organs at risk

Target volumes	Mandatory	Ideal
PTV70 D98%	≥70.0 Gy	-
PTV70 D1cc	≤77.0 Gy	≤74.9 Gy
PTV61.6 D98%	≥61.6 Gy	-
PTV50.4 D98%	≥50.4 Gy	-
CTV_P_	≥70.0 Gy	-
CTV_SVI_ D99%	≥70.0 Gy	-
CTV_ECE_ D99%	≥61.6 Gy	-
CTV_LN_ D99%	≥50.4 Gy	-
Organs at risk		
Rectum D15%	≤74.0 Gy	≤74.0 Gy
Rectum D25%	≤69.0 Gy	≤60.0 Gy
Rectum D35%	≤64.0 Gy	≤50.0 Gy
Rectum D50%	≤59.0 Gy	≤40.0 Gy
Bladder D15%	≤79.0 Gy	≤74.0 Gy
Bladder D25%	≤74.0 Gy	≤60.0 Gy
Bladder D30%	≤69.0 Gy	≤50.0 Gy
Bladder D50%	≤64.0 Gy	≤40.0 Gy
Neck of femur D5%	≤44.0 Gy	-
Small bowel PRV D99%	≤52.0 Gy	-
Small bowel V45Gy	≤195 cc	-
Penile bulb mean dose	-	≤51.0 Gy

### Data collection and analysis

Genitourinary and gastrointestinal toxicity were assessed on a weekly basis during radiotherapy, at 1.5 months and 4.5 months post radiotherapy, then at 6 monthly intervals thereafter. Scoring of toxicity was completed using the Radiation Therapy Oncology Group (RTOG) acute and late radiation morbidity scoring criteria.

Efficacy of treatment will be assessed by biochemical no evidence of disease (bNED) as defined by the ASTRO Phoenix definition (nadir + 2.0 mcg/L) [23]. bNED was and will be assessed at each post-RT review. Treatment efficacy outcome results will be presented at a later date when longer follow-up has been achieved.

Evaluation of compliance with the trial protocol for nomogram-directed target volume delineation, contouring, and dose constraints were conducted after the final patient completed radiotherapy. To demonstrate feasibility, we aimed to achieve ≥90 % protocol compliance rate with each of these parameters.

Up to seven target structures (CTV_P_, CTV_ECE_, CTV_SVA/SVI_, CTV_LN_, PTV70, PTV61.6, and PTV50.4) were generated for each patient according to the protocol outlined in Table 1. At the time of analysis, each patient’s plan was reviewed to determine whether appropriate target structures were treated according to the threshold of ≥15 % risk of involvement as estimated by the MSKCC nomogram. Each target structure was assessed for strict adherence to the contouring protocol by a third party not involved in the original planning process (RW). This assessment was repeated for contouring of OAR.

Dose constraints for target volumes and OAR were assessed according to the objectives listed in Table 2. Values exceeding the ‘mandatory’ limits were termed ‘major variations’. Values in between the ‘mandatory’ and ‘ideal’ limits were termed ‘minor variations’. In all cases, descriptive statistics generated from Microsoft Excel are presented.

Sub-studies examining the use of imaging to predict risk of ADT-induced loss of bone mineral density, and the prognostic significance of circulating tumour cells were completed concurrently using the same patient cohort and are reported on separately [24, 25].

## Results

### Patient characteristics

Twenty eight patients were enrolled onto the trial, of which 27 (96.4 %) completed the planned treatment without unscheduled breaks. The remaining patient was not suitable for treatment due to an acute myocardial infarction prior to radiotherapy. Two patients were enrolled to the trial despite baseline characteristics not fulfilling the inclusion criteria for high risk disease. Median follow-up at the time of analysis was 11.4 months. The patient characteristics are shown in Table 3.

**Table 3:** Baseline patient characteristics

	Median (range)
Age	70.6 years (54.6–78.9)
PSA	12.4 ng/mL (4.0–52.1)
% biopsy cores positive	50 % (25–100)
Gleason Score	Number of patients (percentage)
3 + 4	2 (7 %)
4 + 3	3 (11 %)
4 + 4	3 (11 %)
4 + 5	16 (59 %)
5 + 4	4 (15 %)
T stage	Number of patients (percentage)
T1b	1 (4 %)
T1c	3 (11 %)
T2a	1 (4 %)
T2b	7 (25 %)
T2c	4 (14 %)
T3a	9 (32 %)
T3b	3 (11 %)

### Nomogram use and radiotherapy treatment volumes

The MSKCC nomogram was used to estimate risk of loco-regional spread (ECE, SVI and LNI) both prior to radiotherapy (to direct treatment), and later at the time of data analysis. There was a difference in nomogram outputs between these two time points (Table 4). Student’s t-test demonstrated significant increases for ECE and LNI (both *p* < 0.001), but no change for SVI (*p* = 0.35). If current nomogram outputs were used instead of those obtained prior to RT, 9 of 27 patients would have received different treatment; seven patients using larger volumes and two patients using smaller volumes.

**Table 4:** Nomogram estimates for risk of LRS at pre-radiotherapy and at time of data analysis

	Pre-RT	At analysis	Paired *t*-test
ECE	70.7 % (20.8)	87.1 % (13.8)	*p* < 0.001
SVI	47.0 % (24.1)	43.5 % (24.7)	*p* = 0.356
LNI	32.05 % (27.7)	50.4 % (26.9)	*p* < 0.001

Data is presented as mean (standard deviation)

Two patients with known pelvic lymph node metastases were entered as 100 % risk of LNI

**Fig. 1:**
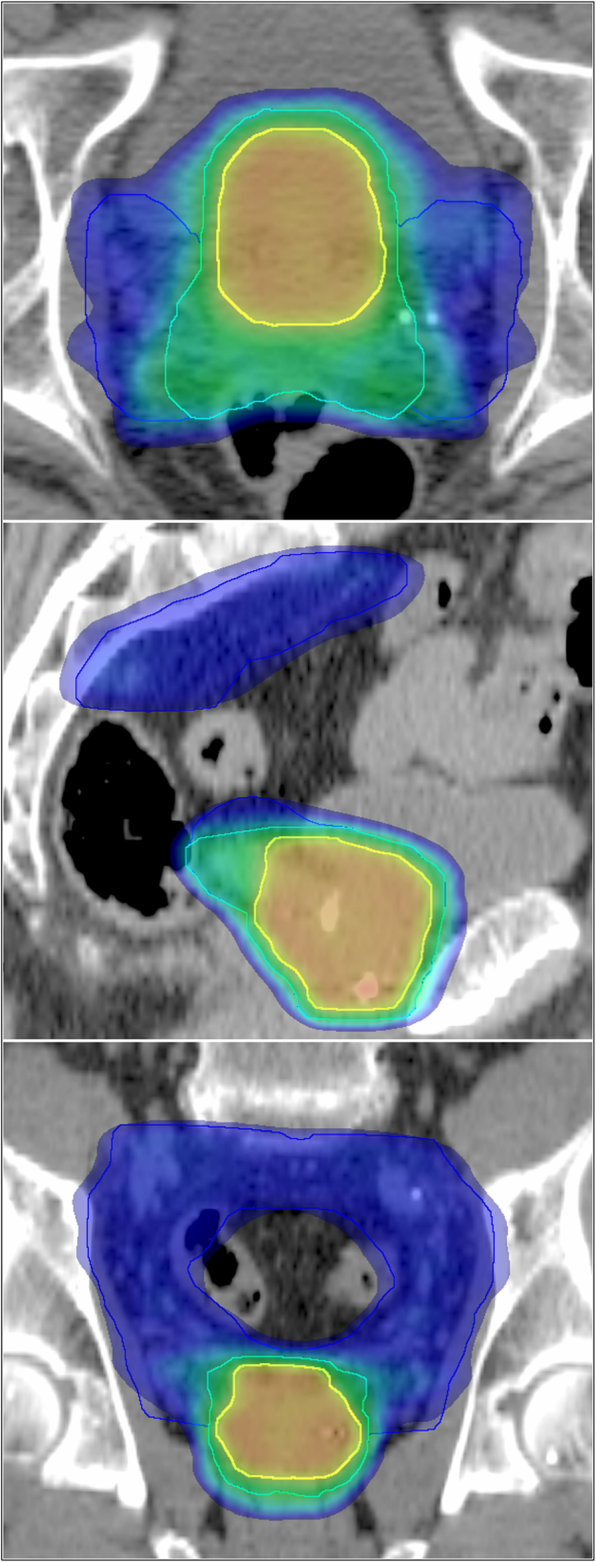
Typical radiotherapy dose distribution. Legend: PTV70 (yellow), PTV61.6 (cyan) and PTV50.4 (dark blue) are displayed with dose colour wash overlay

Radiotherapy target volumes were expanded to account for risk of ECE in 27/27 patients (100 %), SVI in 24/27 (88.9 %), and LNI in 19/27 (70.4 %). An example of the volumes treated to three different dose levels is shown in Fig. 1. Areas at risk of LRS were appropriately included/omitted from treatment according to the study protocol in 98.8 % of cases. However, error in inputting post-ADT rather than pre-ADT PSA into the nomogram for two patients resulted in falsely low estimates of LRS, and the incorrect omission of treatment of both SV and PLN.

### Contouring of target volumes and OAR

The seven target volumes were correctly delineated according to the trial contouring protocol in 94.1 % of cases (160/170). Compliance with protocol for contouring of OAR (rectum, bladder, small bowel) was 70.4 % (57/81), although 22/24 variations were due to small bowel contouring that was incorrect or omitted.

### Dose constraints

Each patient’s radiotherapy plan was assessed for adherence to 5–8 target volume dose constraints and up to 13 OAR dose constraints, dependent upon the volumes treated. Compliance with dose constraints for target volumes was 97.1 % (170/175), with 1.7 % minor variations and 1.1 % major variations. Compliance with dose constraints for OAR was 88.2 % (285/323), with 9.9 % minor variations and 1.9 % major variations.

### Acute toxicity

There was no grade ≥3 genitourinary (GU) or gastrointestinal (GI) acute toxicity observed (Table 5). 20/27 (74.1 %) patients experienced grade 2 GU toxicity at some point during radiotherapy. In all cases this was either increased obstructive or irritative urinary symptoms managed with supportive measures such as Tamsulosin or urinary alkalinisation respectively. 6/27 (22.2 %) patients experienced grade 2 GI toxicity in the form of increased bowel frequency managed with Loperamide.

**Table 5:** Maximal acute toxicity (during radiotherapy)

	Grade 0	Grade 1	Grade 2	≥ Grade 3
Genitourinary	2 (7.4 %)	5 (18.5 %)	20 (74.1 %)	0 (0 %)
Gastrointestinal	12 (44.4 %)	9 (33.3 %)	6 (22.2 %)	0 (0 %)

Data is presented as number of patients (% of total cohort)

## Discussion

We have demonstrated the feasibility of a risk-adjusted radiotherapy treatment protocol that adapts target volume delineation based on nomogram estimates of risk of LRS. This treatment was shown to be technically feasible, clinically practicable, and resulted in acceptable levels of acute toxicity in line with standard of care.

It is important to appreciate the natural patterns of spread of disease when determining target volumes to be treated. A rich surgical pathological literature is available to inform this approach, demonstrating the frequency, and often extent of disease involvement. For example, the risk of SV involvement in patients with T2 disease has been described, as has the fact that in 90 % of such cases disease is confined to the proximal 20 mm of the SV measured along the axis of the structure [4]. It is perhaps noteworthy that in the HNSCC setting, such data regarding pathological risk of loco-regional involvement is deemed appropriate to allow clinical implementation without prospective clinical trial validation [26]. Yet in the prostate radiotherapy setting, clinical trials attempting to quantify the benefit of WPRT continue to be performed (e.g. RTOG 0534 and RTOG 0924). In the era of improved imaging, integration of new systemic agents, and highly conformal radiotherapy, it will be challenging for such studies to definitively answer such questions for all patients, which is part of the reason that most modern protocols simply mandate the extent of elective volume treatment [27].

The twenty-eight treatment hypofractionated radiotherapy regimen used in this study was first described by the Cleveland Clinic [28]. This original protocol has been adapted to form the experimental arm in the RTOG 0415 study, a multi-centre phase III randomized controlled trial examining modest hypofractionation for treatment of favourable risk prostate cancer. Neither of these treatment regimens included elective WPRT. Two separate groups in the US have published their experiences administering conventionally fractionated WPRT concurrently or sequentially with hypofractionated prostate irradiation [16, 29]. Early data regarding biochemical control and toxicity from these four groups have demonstrated encouraging results with the modestly hypofractionated treatment.

The frequency of grade ≥2 acute GU toxicity (74.1 %) observed in this trial was slightly higher than that recorded by the aforementioned studies of McDonald et al. (52 %) and Pollack et al. (approximately 56 %) [16, 29]. This difference may be accounted for by the increased dose to the seminal vesicles (61.6 Gy vs. 56 Gy or 50 Gy respectively) or more likely, a lower threshold for the use of interventions. The increase in toxicity was limited in severity to RTOG grade 2, and it remains to be seen whether this will translate into more meaningful differences in late toxicity. The absence of grade 3 acute toxicity in this study is reassuring and consistent with the published data using similar treatments. The incidence of grade ≥2 acute GI toxicity (22.2 %) was in the same range as the levels seen in the University of Alabama at Birmingham series (37 %) [16]. Their series treated all HRPC men with the same radiotherapy doses to the primary disease and pelvic lymph nodes as in our cohort, and have reported efficacy and late toxicity rates similar to conventional treatment. Our data adds to the literature that supports the feasibility of moderately hypofractionated radiotherapy treating the prostate and pelvis concurrently for men with HRPC.

The question remains as to how best to select patients for radiotherapy volume adaptation. Some guidelines such as from the EORTC recommend using the D’Amico risk stratification. This would probably lead to overtreatment, as some patients designated as high risk actually have very favourable outcomes, illustrating the heterogeneity of such risk groupings [30]. Clinical tools such as the ‘Partin tables’ [31] have analysed historical data from large cohorts of patients undergoing radical prostatectomy to demonstrate the correlation between LRS and prognostic factors such as PSA, GS, and clinical staging. This data could provide an individualized estimate for risk and degree of LRS, which may then be used to adapt the extent of treatment. The use of a web-based nomogram (such as the MSKCC nomogram) allows further refinements to this approach. The clinical tool is widely accessible, simple to use, considers the additional variable of tumour volume, and considers relevant prognostic factors as continuous rather than discrete variables. Furthermore, as the calculations are not completed manually, the underlying algorithm can be sufficiently complex to achieve maximal accuracy. For these reasons, a computer-based nomogram is a powerful tool that facilitates risk-adapted treatment individualization.

There are, however, a number of limitations in using a nomogram in this fashion. First of all, the nomogram is dependent upon historical data that may not be suitable for extrapolation to the current population. Changes in disease epidemiology, staging, and screening practices mean that the effect of prognostic factors may differ between contemporary and historical populations, and the estimates may therefore be inaccurate. A key example of this was the upward migration of Gleason scoring in recent years, partly due to the altered definitions of the core biopsy grading system introduced in 2005 [32]. There is therefore a need to regularly review the applicability of historical results to current populations and update the nomogram algorithms accordingly (which then also prompts the need for external re-validation). The degree to which this affects results is illustrated in the difference in nomogram outputs between the time of planning and analysis (Table 4).

Secondly, most clinical tools used to estimate the risk of LRS in prostate cancer are based upon radical prostatectomy series that employed limited lymph node dissection. It has been demonstrated that standard/limited pelvic lymph node dissections may result in false negative rates for pathological involved nodes of over 50 % compared to extended dissections [33]. Nomogram algorithms that have been derived from this data may therefore generate estimates of LNI that are deceptively low.

Thirdly, there is a danger that data entry errors may result in grossly inaccurate estimates of LRS and incorrect clinical decision-making. For example, a misplaced decimal point, or inputting the post-ADT PSA rather than PSA at diagnosis may alter the nomogram estimates considerably. The latter error occurred twice in our study and resulted in artificially low estimates of LRS and incorrect non-treatment of seminal vesicles and pelvic nodes in these patients. Simple safeguards would prevent such errors from occurring, for example, an observer to verify correct data entry and nomogram use.

Fourthly, there are small sub-groups of prostate cancer patients who are not suitable for nomogram-directed adaptation of treatment. Outcomes for PSA-negative tumours for example are not correctly predicted with current nomograms. This group however represents only a very select subset of patients (1 % or less of total prostate cancer cases) who very often present late with metastatic disease that is not suitable for curative treatment [34]. Neuroendocrine carcinoma of the prostate is another group for which standard prognostic tools are similarly unsuitable.

A further lesson from our experience was appreciating the danger in over-complicating treatment. The novel treatment regimen used in this study involves a number of features that increase its complexity compared to standard practice. These include the use of a nomogram to define risk of LRS and adapt target delineation, protocolised generation of multiple target structures to be treated using up to three different dose levels, and a hypofractionated regimen with many unfamiliar dose constraints. Added complexity is liable to increase the likelihood of errors and protocol non-compliance and must be justified with a benefit to clinical outcomes. We identified a number of examples of this, including rotation of the simulation CT images to contour the proximal seminal vesicles along their axes, or the use of multiple, redundant dose constraints for rectum and bladder. Here, excessive and unfamiliar processes are unlikely to improve outcomes and should be simplified. If additional complexity is value-adding, it may be necessary to implement further safeguards such as peer review of contouring and the use of checklists to maximise protocol compliance.

We have demonstrated feasibility and deliverability of a complex risk-adapted treatment for patients with HRPC. Many future directions are being pursued along similar lines. The use of more extreme hypofractionation coupled with pelvic radiotherapy is increasing, for example in the ‘SATURN’ trial, in which stereotactic radiotherapy treatment is administered over 5 fractions to both the prostate and pelvic lymph nodes. A similar protocol used in the earlier ‘FASTR’ trial however resulted in unacceptable levels of late toxicity, suggesting caution in using such an approach [35].

In contrast, emerging imaging modalities such as PSMA PET are likely to detect early metastatic spread with increased sensitivity, which may reduce the number of at-risk patients with negative staging investigations who are therefore candidates for elective loco-regional treatment. If this does eventuate, however, we would then face the question of how to treat this growing group of patients with early loco-regional or oligometastatic disease, an area where there is again a paucity of evidence to guide management. It is likely that the management of prostate cancer will shift further towards a risk-adapted approach as the results of current trials and integration of new imaging into clinical practice continues.

## Conclusions

We have demonstrated the feasibility of this novel risk-adapted radiation treatment protocol for HRPC. This study has identified key learning points regarding this approach, including the importance of standardization and updating of risk quantification tools, and the utility of an observer to verify their correct use.

## Abbreviations

ADT: androgen deprivation therapy
bNED: biochemical no evidence of disease
CT: computed tomography
CTV: clinical target volume
ECE: extra-capsular extension
ECOG: Eastern Cooperative Oncology Group
GI: gastrointestinal
GS: Gleason Score
GU: genitourinary
HNSCC: mucosal squamous cell carcinoma of the head and neck
HRPC: high risk prostate cancer
IMRT: intensity modulated radiotherapy
LNI: pelvic lymph nodes involvement
LRS: loco-regional spread
MRI: magnetic resonance imaging
MSKCC: Memorial-Sloan-Kettering Cancer Center
OAR: organs at risk
PET: positron emission tomography
PLN: pelvic lymph nodes
PRV: planning organ at risk volume
PSA: prostate specific antigen
PSMA: prostate specific membrane antigen
PTV: planning target volume
RCT: randomized controlled trials
RT: radiotherapy
RTOG: Radiation Therapy Oncology Group
SV: seminal vesicles
SVI: seminal vesicles involvement
WPRT: whole pelvis radiotherapy
